# Direct and indirect costs of long bone fracture nonunions of the lower limb: the economic burden on the German healthcare system

**DOI:** 10.1302/2046-3758.144.BJR-2024-0150.R2

**Published:** 2025-04-09

**Authors:** Tanja C. Maisenbacher, Mika F. Rollmann, Maximilian M. Menger, Niklas R. Braun, Benedikt J. Braun, Steven C. Herath, Fabian Stuby, Andreas K. Nuessler, Tina Histing, Marie K. Reumann

**Affiliations:** 1 Department of Trauma and Reconstructive Surgery, Eberhard Karls University Tuebingen, BG Klinik Tuebingen, Tuebingen, Germany; 2 Siegfried Weller Institut für Unfallmedizinische Forschung, Eberhard Karls University Tuebingen, BG Klinik Tuebingen, Tuebingen, Germany; 3 Department of Trauma and Reconstructive Surgery, BG Unfallklinik Murnau, Murnau, Germany

**Keywords:** Nonunion, Fracture, Costs, Socioeconomic burden, Indirect costs, lower limb, long-bone fractures, fractures and nonunions, Orthopaedic Trauma, orthopaedic surgery, retrospective cohort study, Mann-Whitney U test, comorbidities, bone nonunions, fracture of the tibia/fibula

## Abstract

**Aims:**

Fracture nonunion represents a major complication in orthopaedic surgery, occurring in 5% to 10% of fracture patients. Fracture nonunions are associated with pain and loss of function, and lead to a substantial socioeconomic burden. The present retrospective cohort study analyzed direct and indirect costs and length of hospital stay, number of surgical procedures, and hospital (re-)admissions of nonunion patients.

**Methods:**

Data from 18- to 65-year-old patients surgically treated for lower limb fractures and nonunions in a German level I trauma centre between 2012 and 2018 were analyzed. A total of 193 patients with nonunion were included, and 2,511 patients with fractures served as the control group. Direct costs were calculated using reimbursement according to the diagnosis-related group (DRG). Indirect costs were calculated including daily sickness allowance and productivity loss.

**Results:**

The median healing time of nonunion patients was 45 weeks. Treatment expenses showed a 2.6-fold increase in direct costs, a 3.3-fold increase in indirect costs, and a 3.3-fold increase in total costs for nonunion patients compared to the control group. As every patient with a nonunion suffered from a fracture prior to nonunion treatment, costs were calculated by adding the median direct costs of €10,487 (IQR 9,173 to 15,262), median daily sickness allowance of €23,046 (IQR 14,892 to 36,264), median productivity loss of €85,714 (IQR 60,949 to 126,650), and median total socioeconomic burden of €123,334 (IQR 88,630 to 176,329).

**Conclusion:**

Nonunions not only pose a significant burden on the injured individual and on healthcare systems, but also have a substantial socioeconomic impact. High direct and indirect costs illustrate that healing complications need to be detected and addressed as early as possible.

Cite this article: *Bone Joint Res* 2025;14(4):1–10.

## Article focus

- Nonunions pose a huge burden on socioeconomic systems.
- Direct and indirect costs were analyzed, as well as the length of hospital stay, number of surgical procedures, and hospital (re-)admissions resulting from post-traumatic long bone nonunions of the lower limb.

## Key messages

- Development of a fracture nonunion leads to a 3.3-fold increase in overall costs.
- Costs are mainly driven by indirect costs.

## Strengths and limitations

- By including both direct and indirect costs, a realistic picture of the overall cost is given.
- Some costs, such as outpatient treatment costs, and details of the fracture control group could not be assessed.

## Introduction

Fracture nonunion represents a major complication in orthopaedic surgery. Despite substantial progress in (pre)clinical research on bone healing, 5% to 10% of all fractures still result in nonunion.^1,2^ Nonunion rates are reported to be as high as 14% for certain lower limb fractures depending on the location and treatment characteristics.^3^ The treatment of nonunion is complex, expensive, and highly challenging, as each patient requires an individual treatment plan.^4,5^

The ‘diamond concept’^6,7^ of fracture healing describes a variety of factors enabling successful bone regeneration: 1) availability of osteogenic cells and 2) osteoinductive mediators, 3) a suitable osteoconductive matrix,^8-10^ 4) sufficient mechanical stability,^11^ and 5) adequate vascularization.^12^ Both biological and mechanical factors need to be addressed in the management of nonunions, e.g. by autologous bone grafting^8,10^ and a biomechanically stable re-osteosynthesis.^11^ Moreover, individual risk factors and underlying comorbidities must be assessed, including smoking and alcohol consumption,^13,14^ obesity, malnutrition, osteoporosis, and diabetes mellitus.^15-17^ In many cases, the treatment of fracture nonunion requires surgical revision, and is usually associated with additional hospitalization and a prolonged rehabilitation process. Nonunions represent a rare condition; thus, they are usually treated in specialized tertiary care institutions by well-trained specialists in the field,^18,19^ as a delay in diagnosis leads to poor outcome.^20^

Accordingly, nonunions not only result in considerable pain and loss of function for the individual, but are also associated with a substantial economic burden on healthcare systems.^21^ Several studies have been conducted on certain areas of health expenditure caused by nonunions, including the comparison of different treatment options, the costs of inpatient treatment, and the cost attributable to complications or cost-effectiveness based on quality-adjusted life-years (QALYs).^1,22-27^ In the USA, a twofold increase in costs associated with development of a fracture nonunion was shown.^24^ Data from an Australian Registry showed that 8.1% of patients require hospital readmission due to fracture healing complications, leading to median direct costs of AUD $14,957.^25^ Within the German healthcare system, two review articles addressed costs for nonunion treatment, but no detailed numbers were presented.^1,26^ To our knowledge, however, the expenditure caused by nonunion focusing on direct as well as indirect costs has yet to be precisely quantified.

Therefore, the aim of the present study was to analyze direct and indirect costs, the length of hospital stay, and the number of surgical procedures and hospital (re)admissions resulting from long bone nonunions of the lower limb following fracture compared to a control group of patients with fractures and regular bone healing.

## Methods

### Nonunion and fracture control group

All patients with delayed union, malunion, and nonunion following a long-bone fracture of the lower limb, and requiring surgical treatment for nonunion in a German level I trauma centre between 2012 and 2018, were included. A nonunion was defined as follows: radiological evaluation according to the Radiographic Union Score for Tibia (RUST) (number of cortices showing callus formation in anteroposterior and lateral view of radiographs),^28,29^ clinical signs of nonunion according to clinical reports (pain, loss of weightbearing),^1^ and at least six months past initial fracture.^1^

Only patients between the age of 18 and 65 years were included, since the official retirement age in Germany at the time of data collection was 65 years.^30^ A substantial part of the indirect costs of fracture nonunion treatment is attributable to daily sickness allowance paid by health insurance providers, and the loss of productivity, which is only applicable to the working population. Patients undergoing conservative treatment of their fracture nonunion as well as nonunion after osteotomies or bone tumours were excluded.

Patients requiring surgical treatment of long bone fractures (International Classification of Diseases (ICD) - codes S72 and S82 (excluding S82.0 – patella fractures))^31^ of the lower limb served as the control group, following the same exclusion and inclusion criteria.

### Study design

**Fig. 1:**
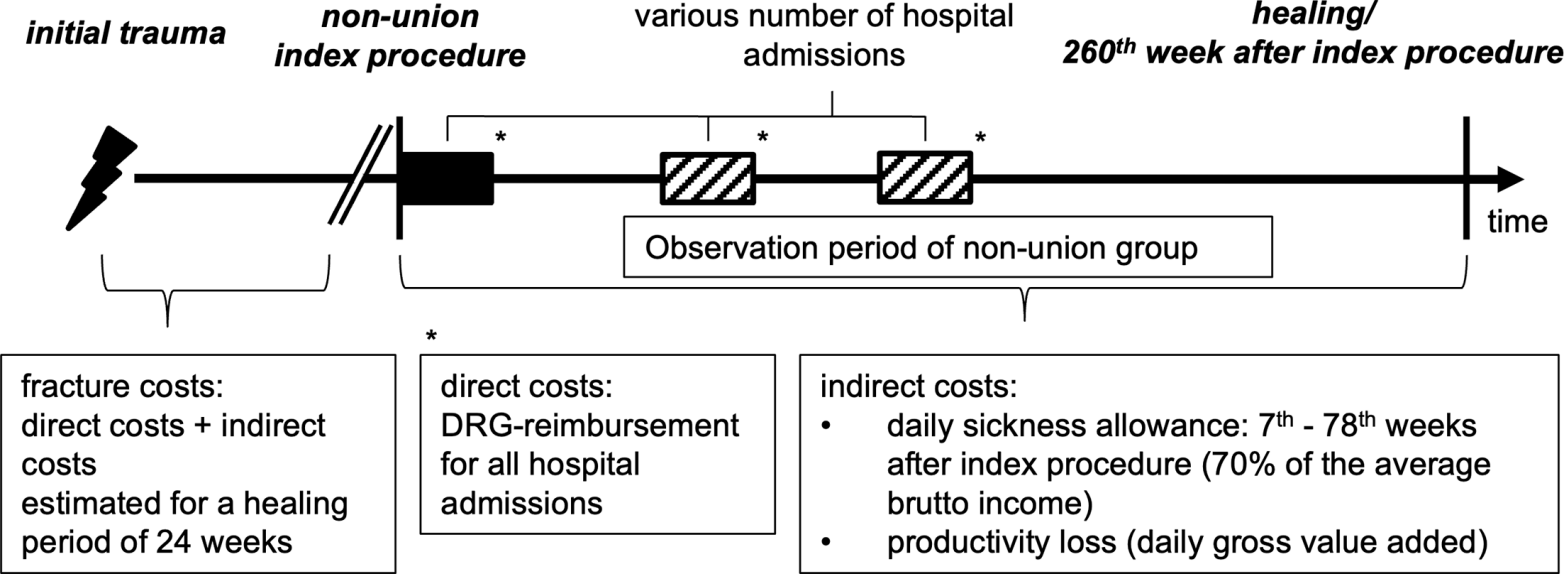
Study design. Study initiation was defined as the day of the first fracture nonunion surgery indicated as ‘index procedure’. The endpoint was defined as the day of diagnosed and documented bony consolidation. If this did not occur, the maximum observation period was set at 260 weeks. Direct costs represent diagnosis-related group (DRG) reimbursement. Indirect costs were defined as daily sickness allowance to be reimbursed by health insurances, which is paid between the seventh and 78th week in the German healthcare system, plus the loss of work productivity.

The first surgical procedure to treat the nonunion was defined as the index procedure and beginning of the observation period (Figure 1). The observation period ended on the day when consolidation was diagnosed, or treatment reached the 260^th^ week after index procedure. Hospitals in the German healthcare system are compensated per case through a yearly adjusted case mix approach according to the diagnosis-related group (DRG).^32^ The DRG is primarily determined by the corresponding ICD codes and the specific Code for Operations and Procedures (German: Operationen und Prozedurenschluessel - OPS).^33^

### Cost analysis

The case mix approach for the relevant year(s) and DRGs were used to calculate direct costs. Supplementary Table i details German healthcare-related technical terms, and explanations for costs and reimbursement.

Indirect costs are defined by daily sickness allowance paid by the health insurance providers after the index procedure plus the costs for loss of productivity. Loss of productivity was calculated according to the example of the German federal office for occupational safety and health by using the average gross value added per day.^34^ The daily sickness allowance is determined by the average gross income in Germany published each year by the German federal office of statistics.^35^ German health insurance providers pay 70% of the gross income for each patient as daily sickness allowance, starting from week 7 after the first day of sick leave to week 78.

The exact healing time of each patient in the control group of fracture patients was not available. We assumed an average time period of 20 weeks for return to work to be able to make a comparison with the incurring costs for the study group.^36-38^ Hence, the indirect costs were calculated for a length of 20 weeks as described above.

The Ethical Committee of the Eberhard Karls University Tübingen provided ethical approval for this study.

### Statistical analysis

Descriptive statistical analysis was performed to display the basic demographic and clinical characteristics of the selected patients. Mann-Whitney U test was performed to test significant differences between both groups. Spearman correlation was used to analyze possible correlations of continuous variables. To identify the distribution of both groups, Anderson-Darling test was performed. All statistical analyses were conducted using “JMP” version 15.0.0 (SAS Institute, USA). A p-value < 0.05 was considered to indicate significant differences.

## Results

### Characterization of patient cohorts

**Fig. 2:**
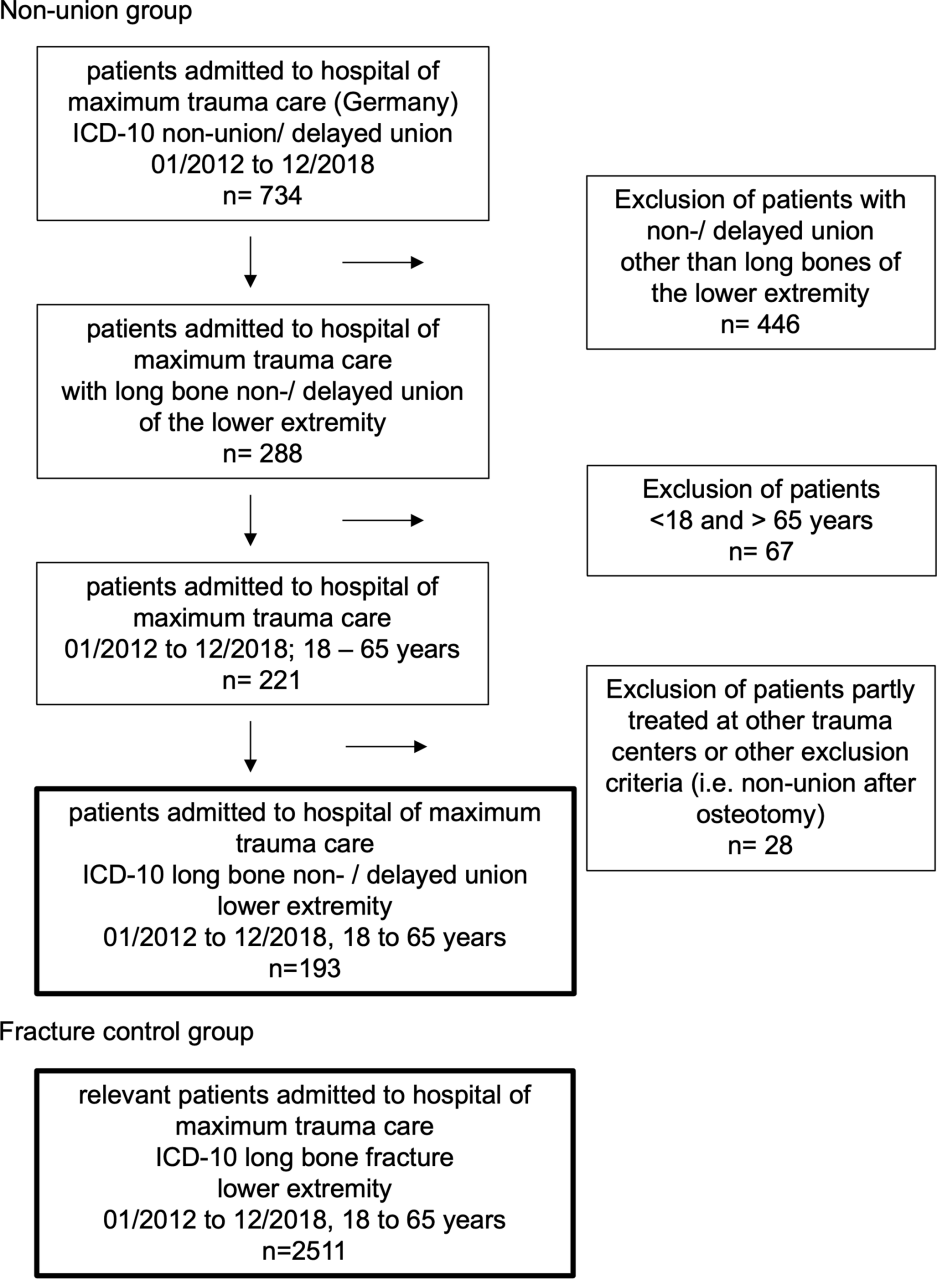
Case selection flowchart. From 2012 to 2018, 734 patients with fracture nonunion were admitted to our level 1 trauma hospital. All anatomical locations other than the long bones of the lower limb were excluded (n = 446). A total of 67 patients aged under 18 and over 65 years were excluded. A total of 28 patients did not receive full treatment in our hospital, or were excluded based on other exclusion criteria such as nonunion after osteotomy. In summary, 193 patients could be identified for further investigation. According to inclusion and exclusion criteria, 2,511 patients with long bone fractures of the lower limb were treated during the same period and served as the control group. ICD, International Classification of Diseases.

Based on the inclusion and exclusion criteria, 193 patients with long bone nonunion of the lower limb were identified (Figure 2). Of those patients, 24.9% were female (n = 48) and 74.1% male (n = 145). In total, 2,511 patients were treated for long bone fractures of the lower limb within the observation period serving as control group. Of those patients, 47.0% were female (n = 1,181) and 53.0% male (n = 1,330). The median age of the patients within the nonunion group was 47.4 years (IQR 37.0 to 52.4) at the time of the index procedure, whereas median age of the control group was 48.0 years (IQR 33.0 to 56.0). Regarding anatomical location in the nonunion group, 37.8% were located at the femur (n = 73) and 62.2% at the lower leg (n = 120). Within the control group, 22.7% of patients suffered from a femur fracture (n = 571) and 77.3% from a fracture of the tibia/fibula (n = 1,940). Statistical analysis showed that there were significantly more males than females in both groups (Table I) (p < 0.001, Mann-Whitney U test). Data revealed no significant differences between the overall age of nonunion and fracture control group, however female nonunion patients were significantly younger than female fracture patients (p < 0.001, Mann-Whitney U test). No significant differences regarding age could be detected in the male cohorts.

**Table I.** Comparison of the nonunion and fracture control group.

Parameter	Fracture control group	Nonunion group
Number of patients	2,511	193
**Sex, n (%)**		
Female	1,181 (47.0)*	48 (24.9)
Male	1,330 (53.0)†	145 (74.1)†
**Median age, yrs (IQR)**		
All patients	48.0 (33.0 to 56.0)	47.4 (37.0 to 52.4)
Female	51.0 (40.0 to 58.0)*	46.1 (35.9 to 50.8)
Male	45.0 (30.0 to 54.0)	47.5 (37.8 to 52.9)
**Anatomical location, n (%)**		
Femur	571 (22.7)	73 (37.8)
Lower leg	1,940 (77.3)†	120 (62.2)†

* Horizontal comparison (p < 0.001, Mann-Whitney U test).

† Vertical comparison (p < 0.001, Mann-Whitney U test).

Concerning anatomical location, results indicated significantly more lower leg fractures/nonunions than femur fractures/nonunions in both groups (p < 0.001, Mann-Whitney U test).

### Analysis of nonunion treatment

**Fig. 3:**
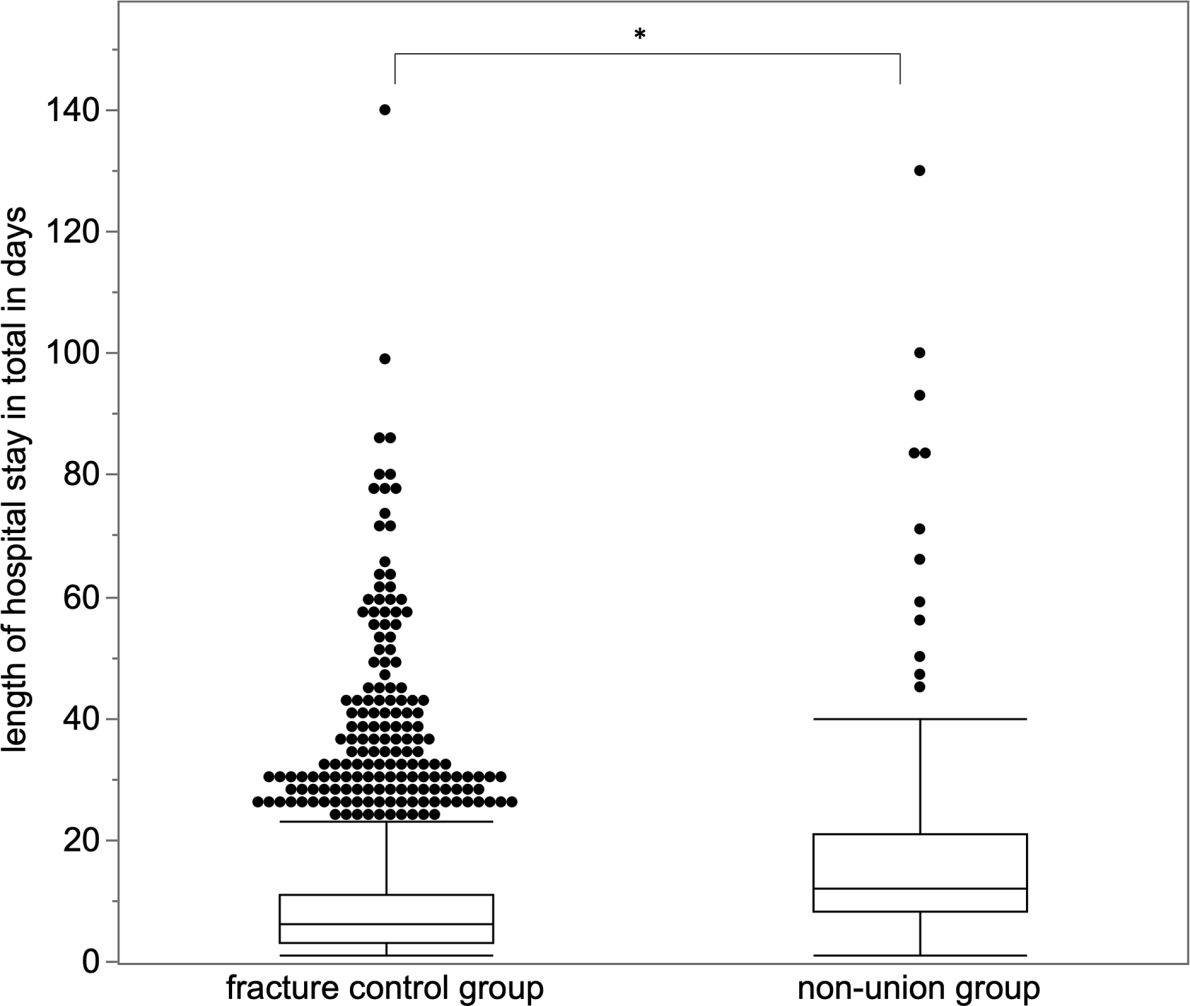
Length of inpatient treatment. Fracture nonunion patients had significantly more days of inpatient treatment in total than fracture control group patients (p < 0.001*,* Mann-Whitney U test*)*. Patients of the fracture control group had a median length of hospital stay of six days (IQR 3 to 11). The inpatient treatment of fracture nonunion patients added up to a median of 12 days (IQR 8 to 21) in total.

The median time from the index procedure to bone healing was 45 weeks (IQR 25.9 to 75.9) per patient. Accordingly, the median time for daily sickness allowance claimed was 39 weeks (IQR 19.9 to 69.9), as this is not paid for the first six weeks of sick leave. The majority (94.8%, n = 183) of patients required one to three hospital admissions until the documented bone consolidation. Overall, 3.1% (n = 6) of patients were admitted to hospital between four and six times, and 2.1% (n = 4) required seven to nine admissions. The number of hospital admissions per patient was closely related to the number of surgical interventions: 92.7% (n = 179) of patients had one to three surgical interventions, 4.7% (n = 9) required four to six, and 2.6% (n = 5) up to 12 surgeries until healing of the fracture nonunion could be achieved. The median length of overall hospital stay per patient was 12 days (IQR 8 to 21); the median length of stay per admission was ten days (IQR 7 to 14) (Table II). The median length of inpatient treatment per patient in total was significantly longer in the nonunion group compared to the fracture control group (Figure 3).

**Table II.** Data related to the treatment of nonunions (n = 193).

Parameter	Nonunion value
Median time to heal, wks (IQR)	45 (25.86 to 75.86)
Median claim on daily sickness allowance, wks (IQR)	39 (19.86 to 69.86)
**Hospital admissions per patient, n (%)**	
1 to 3	183 patients (94.8)
4 to 6	6 patients (3.1)
7 to 9	4 patients (2.1)
Median number of admissions per patient (IQR)	1 (1 to 2)
**Number of surgical interventions (%)**	
1 to 3	179 patients (92.7)
4 to 6	9 patients (4.7)
7 to 12	5 patients (2.6)
Median number of surgical interventions (IQR)	1 (1 to 2)
Median length of hospital stay, days (IQR)	12 (8 to 21)
Median days of hospital stay per admission (IQR)	10 (7 to 14)

**Fig. 4:**
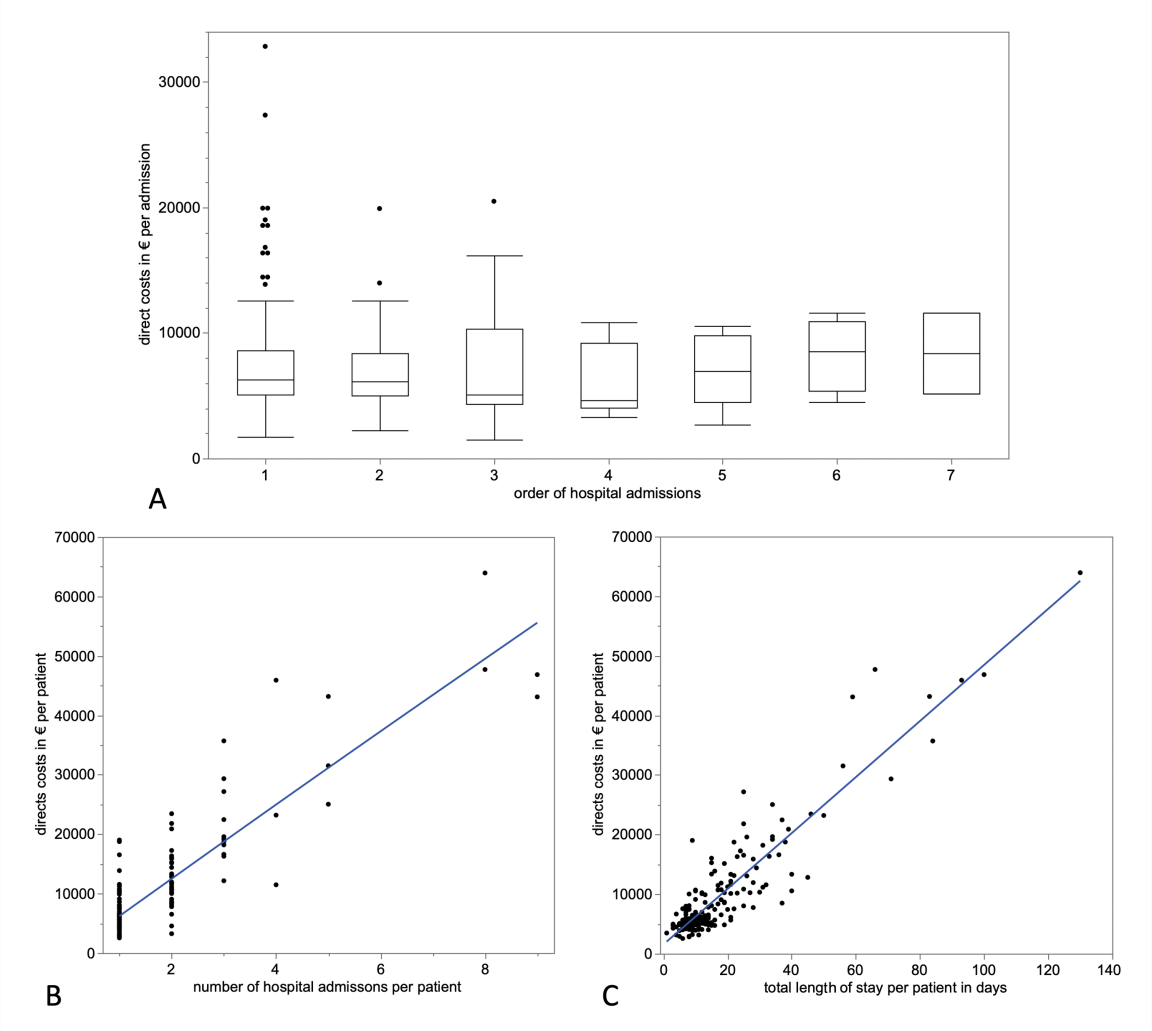
Impact of the length of inpatient treatment on direct costs. a) There were no significant differences in direct costs across all hospital admissions. All fracture nonunion patients required at least one hospital admission (n = 193). A total of 60 patients required two hospital admissions, 21 patients three admissions, nine patients four admissions, seven patients five admissions, four patients six admissions, and three patients seven admissions, until healing of the nonunion could be achieved or treatment ended. b) There was a significant correlation between the number of hospital stays and direct costs (€) (p < 0.001, r^2^ = 0.78*)*. c) Accordingly, length of hospital stay correlated significantly with direct costs (€) (p < 0.001, r^2^ = 0.84).

Data analysis showed no significant differences in the costs of the single hospital stays (Figure 4a). However, the accumulation of hospital stays for patients with fracture nonunion who require several inpatient treatments showed a strong and significant correlation to direct costs (Figure 4b). Consequently, analysis of total length of hospital stays in days in the nonunion group depicted a strong and significant correlation to direct costs (Figure 4c). Thus, direct costs significantly increased in patients with fracture nonunions with the number of hospitals stays and thereby days of inpatient treatment.

### Direct and indirect costs of nonunion and control group

Neither direct costs nor indirect costs of fractures or fracture nonunions were normally distributed (p < 0.001, Anderson-Darling test).

The median direct costs for the control group of patients with long-bone fractures of the lower limb reached €4,070. The median daily sickness allowance calculated by using 20 weeks of sick leave was at €6,158, and the economic loss, defined as the loss of productivity, at €26,648 per patient (Table III).

**Table III.** Cost expenditure of long-bone nonunion of the lower limb.

Parameter	Fracture control group	Nonunion group	Nonunion group including fracture treatment
Median direct costs, € (IQR)	4,070	6,417 (5,103 to 11,192)	10,487 (9,173 to 15,262)
Median daily sickness allowance, € (IQR)	6,158	16,888 (8,734 to 30,106)	23,046 (14,892 to 36,264)
Median loss of productivity, € (IQR)	26,648	59,066 (34,301 to 100,002)	85,714 (60,949 to 126,650)
Median socioeconomic burden, € (IQR)	37,909	85,425 (50,721 to 138,420)	123,334 (88,630 to 176,329)

As all fracture nonunion patients suffered from a post-traumatic nonunion where nonunion follows treatment of a fracture, costs for fracture and nonunion were added to reach information on costs for fracture nonunion overall. Comparing nonunion with fracture control group, there were significant differences in direct costs, indirect costs, and overall socioeconomic burden (p < 0.001, Mann-Whitney U test). Comparing overall costs (fracture plus fracture nonunion treatment) with fracture control group data showed significant differences for direct, indirect, and overall costs (p < 0.001, Mann-Whitney U test).

**Fig. 5:**
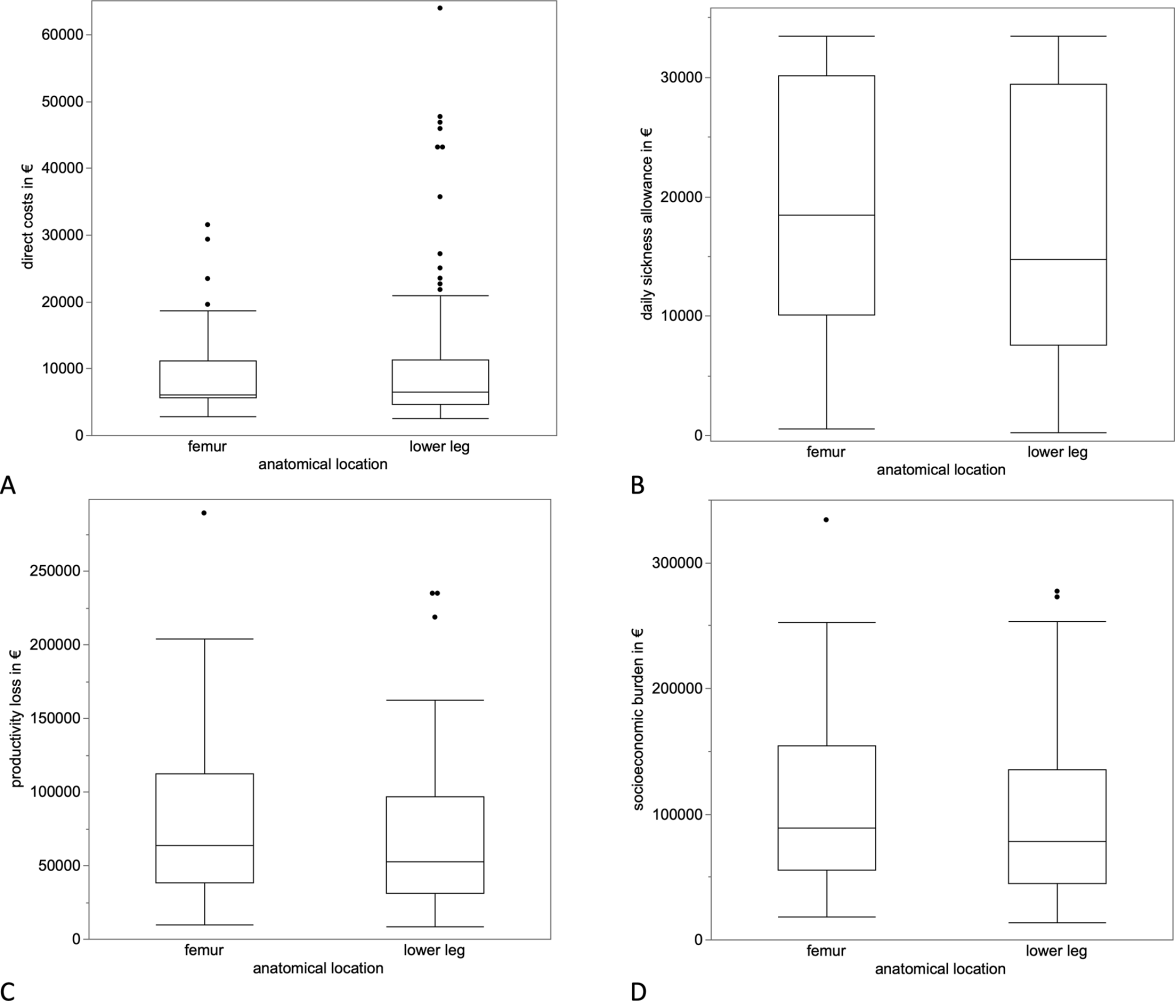
Cost differences in different anatomical locations. a) There were no significant differences in direct costs of fracture nonunion patients concerning the anatomical location (p = 0.120). The median direct cost caused by femoral fracture nonunions was €6,119 (IQR 5,652 to 11,192), and the median of the fracture nonunions located at the lower leg was €6,543 (IQR 4,641 to 11,273). Neither b) median daily sickness allowance (€18,495 (IQR 10,078 to 30,106)) (p = 0.170) nor c) median productivity loss (€63,618 (IQR 38,535 to 112,687)) showed a significant difference in costs (p = 0.100). d) Accordingly, total costs (p = 0.120, all Mann-Whitney U test) did not depict significant differences. The median total costs caused by fracture nonunion located at the femur were €88,802 (IQR 55,205 to 154,610) and €78,475 (IQR 45,187 to 135,546) for the lower leg.

The median direct cost for the treatment of fracture nonunion was €6,417 (IQR 5,103 to 11,192) per patient (Table III). Looking at indirect costs of nonunions, median daily sickness allowance per patient was €16,888 (IQR 8,734 to 30,106). The median economic loss per patient was €59,066 (IQR 34,301 to 100,002) (Table III). There were no significant differences regarding direct, indirect, and overall costs concerning anatomical location (Figure 5). In the nonunion group, comorbidities (diabetes mellitus and cardiovascular diseases) and smoking behaviour of the patients were analyzed, but did not lead to a significant increase in costs (smoking: p = 0.699, cardiovascular diseases: p = 0.731, diabetes mellitus: p = 0.572, Mann-Whitney U test). No correlation between increasing BMI and increasing costs could be found (p = 0.505, *r*^2^ = 0.0002, Spearman correlation).

Assuming that every nonunion patient suffered from a fracture prior to the need for nonunion surgical treatment, the total treatment costs (treatment cost for fracture + treatment costs for nonunion) rose even higher: adding the costs of fracture and nonunion treatment led to median direct costs of €10,487, median daily sickness allowance of €23,046, median productivity loss of €85,714, and median total socioeconomic burden of €123,334.

Comparing both groups' results in the total costs for nonunion showed significantly higher costs for fracture plus nonunion treatment in comparison to costs caused by fracture treatment only (p < 0.001, Mann-Whitney U test). Similarly, the median direct costs and indirect costs of the nonunion group were significantly higher than the costs of the control group (p < 0.001) (Table III). The nonunion group showed a 2.6-fold increase in direct costs, a 3.3-fold increase in indirect costs, and a 3.3-fold increase in total costs compared with the control group (Table III).

## Discussion

Nonunions not only pose a huge burden on the injured individual and on healthcare systems, but also have a substantial socioeconomic impact. Our study revealed that the median time of nonunion healing was 45 weeks, and that treatment expense showed a 2.6-fold increase in direct cost, a 3.3-fold increase in indirect cost, and a 3.3-fold increase in total cost for nonunion patients compared to fracture control group patients. The majority of studies analyzing the cost of nonunion treatment focus on direct health expenditure only, and therefore did not capture the full socioeconomic impact.

As shown in a previous study, the majority of fractures in the working age population in Germany occur in males.^39^ Similarly, the rate of nonunions is higher in males compared to females, with most nonunions presenting in the lower leg as opposed to the femur.^40-42^ In line with this, there were significantly more males than females in both the nonunion group and the fracture control group in our cohort. Additionally, our data showed a larger number of fractures/nonunions in the lower leg than in the femur. Those differences in sex and anatomical location did not make a difference regarding direct costs, as they have been analyzed per patient.

The reimbursement based on the DRG system varies considerably between countries, thereby limiting comparability to other health systems.^43^ Ekegren et al^25^ gathered data of fractures and healing complications from the Victorian Orthopaedic Trauma Outcomes Registry in Australia, analyzing inpatient (direct) costs. Their calculation identified a median of AUD $14,957 (the equivalent of €10,904) of direct costs for patients with fracture healing complications. These patients required up to three hospital admissions with a median length of stay of three days per admission. Direct costs of nonunion treatment in Australia were higher when compared to our data in Germany. However, the number of readmissions in nonunion patients was similar. Inpatient treatment of most of our nonunion patient cohort ranged between one and three admissions. Assuming only one hospital stay for a patient in the fracture control group versus some nonunion patients requiring up to nine hospital admissions illustrates that treatment of nonunion incurred significantly more inpatient treatment days.

According to our data, direct costs for nonunion treatment were 2.6-times higher compared to costs of treatment of fractures. In line with these findings, Antonova et al^24^ found similar results in the USA where the costs for nonunion treatment were 2.2-times higher with USD $25,555.97 (€18,656) for tibial nonunion treatment versus $11,686.24 (€8,531) for treatment of tibial fractures. The difference in costs between Germany and the USA might be due to differences in healthcare systems and reimbursement of health insurance. Furthermore, the average length of hospital stay is considerably longer in Germany compared to the USA, which may also have an impact on differing direct costs.^44^

Kanakaris and Giannoudis^45^ reviewed costs of the treatment of long bone nonunion and found only nine relevant studies between 1994 and 2006. Additionally, they performed a theoretical calculation of a best-case scenario of an aseptic fracture nonunion with only one treatment needed, and revealed overall costs of £17,200 for femoral fracture nonunions and £16,330 for tibial nonunions. The number of hospital admissions in our cohort ranged from one to nine with a range in total costs of €13,729 to €333,976, demonstrating comparable treatment costs as there is a notable increase in costs with the number of hospital admissions.

Calculation of health expenditure of nonunion treatment should not only include direct costs, but also consider indirect costs, especially when trying to point out the socioeconomic impact. Our data show that an increase in costs comparing fracture and nonunion treatment was mainly due to indirect costs, which were 3.3-times higher for nonunions than the indirect costs of fracture treatment. Alt et al^23^ compared the difference in cost between Gustilo-Anderson III tibial fractures treated with or without rhBMP-2 in a multicentre study setting in Germany, France, and the UK, and found a cost reduction of €9,270 per case treated with rhBMP-2 due to a shorter healing period and subsequent lower indirect costs.

In addition to trauma-related risk factors like open fractures,^24^ a number of comorbidities such as diabetes mellitus, osteoporosis, obesity, cardiovascular diseases, and lifestyle factors are clearly associated with the development of a nonunion.^15,16,26^ Hence, the primary treatment of fractures should be planned meticulously and with great expertise. Risk factors for nonunion should be identified early in fracture treatment and addressed accordingly to prevent complications.

However, if fracture nonunion has already occurred, the underlying factors for its development must be considered and treatment has to be planned accordingly to shorten the healing time.^40^ Further research is urgently needed to identify factors leading to prolonged healing times or the need for multiple surgical interventions, in order to reduce the burden on the individual as well as healthcare systems and society.

Although our analysis comprehensively included direct as well as indirect costs, we note some limitations to our study. Outpatient costs, such as physiotherapy, the expenses for homecare, medication, medical aids and appliances, mental healthcare, the loss of productivity of relatives serving as caregivers, or rehabilitation costs, vary widely and cannot be easily assessed. The outpatient treatment costs of, for example, appointments for x-ray controls could not be included. Due to the long course of bone healing, it can be estimated that nonunion patients consult outpatient clinics more often than fracture patients, leading to an even larger difference in overall costs. Additional intangible costs, such as psychological strain or lack of social contact caused by long healing period, were also not possible to consider.

Another limitation is the estimated return-to-work period of 20 weeks for the fracture control group. Available literature concerning the time course of incapacity to work after a long bone fracture is limited. Dehoust et al^36^ found that patients suffering from tibial plateau fractures required a median time to recovery and return to work of 24 to 28 weeks, depending on the fracture pathology. Another study supported these findings by giving a median of 120 days (17 weeks).^37^ Median return-to-work periods after open wedge high tibial osteotomies were reported to be 2.6 months (11 weeks), which can be expected as an approximate healing time for uncomplicated fractures without joint involvement.^38^

Furthermore, the time course between the estimated 20 weeks for potential healing of a fracture and the first surgical intervention of the nonunion needs to be considered. Many patients were not initially treated in our hospital. It can be assumed that in some cases, more than 20 weeks elapsed from the initial trauma to the diagnosis of a nonunion and the first surgical intervention. Diagnosis of delayed or nonunion is known to be a long and complicated process.^1^ Most of the patients did not return to work within this time, thus our reported costs should be taken as a conservative calculation of the true cost.

In summary, the median overall costs for the treatment of a long bone nonunion of the lower limb, including the estimated costs for the treatment of the initial trauma, add up to €123,334 per case in the German healthcare system. The costs were mainly driven by indirect costs. The development of a nonunion after fractures causes a 3.3-fold increase in overall costs. To reduce these costs for treatment of nonunions in future, early detection of delayed healing or healing failure is necessary, as well as an early, adequate, and successful treatment strategy.

## Data Availability

The data that support the findings for this study are available to other researchers from the corresponding author upon reasonable request.
